# Age-related changes in HPT axis sensitivity and their associations with metabolic and cardiovascular health: a cross-sectional, Chinese nationwide study

**DOI:** 10.3389/fendo.2026.1857191

**Published:** 2026-09-09

**Authors:** Lei Zhao, Runqing Mu, Xin Zhang, Shuo Wang, Min Zhao, Hong Shang

**Affiliations:** Department of Laboratory Medicine, National Clinical Research Center for Laboratory Medicine, The First Hospital of China Medical University, China Medical University, Shenyang, China

**Keywords:** aging, cardiovascular risk, Metabolic syndrome (MetS), thyroid hormone, thyroid feedback quantile-based index (TFQI)

## Abstract

**Background:**

The regulation and secretion of hormones in the endocrine system change with aging. While these changes may influence age-related disease risks, the role of hypothalamic-pituitary-thyroid (HPT) axis sensitivity in metabolic and cardiovascular health remains unclear.

**Methods:**

This study enrolled 13,646 participants (6,221 males, 7,425 females). Metabolic (BMI, SUA, FPG, lipoproteins) and cardiovascular (ECG, CK-MB, NT-proBNP) indices were measured. Multivariable logistic regression adjusted for age (per 10-year increment), sex, and outcome-specific confounders. Nonlinear relationships between age and TFQI were analyzed using restricted cubic splines (RCS).

**Result:**

RCS analysis revealed a significant age-dependent decline in TFQI-fT3 values, with an inflection point at 48 years (p<0.001); TFQI-fT4 showed a similar but attenuated trend. For metabolic outcomes, increased fT3 sensitivity (TFQI-fT3 Q1) was associated with lower MetS risk in older adults (>48 years) (OR 0.79, 95% CI 0.65-0.97, p=0.023), whereas no significant association was observed in younger adults (≤48 years) (all p>0.05). For cardiovascular outcomes, higher fT3 sensitivity was associated with reduced risk of abnormal ST segments in both age groups (younger: OR 0.57, 95% CI 0.32-0.97, p=0.046; older: OR 0.68, 95% CI 0.48-0.95, p=0.027). Conversely, decreased fT4 sensitivity (TFQI-fT4 Q4) specifically increased abnormal ST-segment risk in older adults (OR 1.43, 95% CI 1.04-1.96, p=0.025), with no significant effect in younger adults (OR 1.11, 95% CI 0.65-1.82, p=0.693).

**Conclusion:**

Enhanced HPT axis sensitivity to fT3 increased with age (inflection point at 48 years in this study) and was associated with a lower prevalence of metabolic syndrome (in >48-year-olds) and ST-segment abnormalities (all adults).

## Introduction

Thyroid hormones play a pivotal role in regulating metabolism and the cardiovascular system. Abnormal levels of triiodothyronine (T3) and thyroxine (T4) are linked to type 2 diabetes (T2DM), dyslipidemia, nonalcoholic fatty liver disease (NAFLD), and atrial fibrillation (1–4). Emerging evidence suggests that even within reference ranges, variations in thyroid hormone levels associate with metabolic and cardiovascular impairments (5, 6). The Penn Heart Study demonstrated that individuals with high-normal thyroid-stimulating hormone (TSH) (4.51-19.99 mIU/L) and normal fT4 had an increased risk of atrial fibrillation (AF) compared to those with euthyroidism (hazard ratio 1.82, 95% CI 1.27-2.61) (7), highlighting the clinical relevance of subtle thyroid alterations.

Thyroid function varies with age. Aging population often exhibit decreased free serum T3 levels and elevated serum TSH levels, reflecting age-related changes in thyroid regulation (8). Among western populations aged ≥70 years, the prevalence of TSH levels of 4.5 mIU/L or higher is approximately 15% (9), and this prevalence reaches 20% (10) in the Chinese population. Meta-analyses indicate that significantly elevated TSH levels (10 mIU/L or greater) in the elderly are associated with cardiovascular morbidity and all-cause mortality (11, 12). However, moderately elevated TSH levels (4.5-7.0 mIU/L) have not shown a statistically significant impact on aging and disease development (13, 14). This paradox suggests that conventional thyroid markers may not fully capture age-related physiological adaptations.

The hypothalamic-pituitary-thyroid (HPT) axis maintains thyroid hormonal balance through feedback loops. Analyzing combined indices rather than single measurements can provide new insights into thyroid function and metabolism. Central and peripheral thyroid sensitivity can be reflected by various indicators. However, existing studies on HPT sensitivity present three critical gaps: (1) inconsistent findings across euthyroid and hypothyroid populations (15–17); (2) limited data on age-dependent sensitivity changes; and (3) unclear cardiovascular implications. To address these unclear questions, we conducted a cross-sectional analysis of 13,730 adults (aged 18–92 years), systematically evaluating how HPT sensitivity to thyroid hormones varies with age and associates with metabolic/cardiovascular risk stratification.

## Methods

### Study population

This study is a cross-sectional analysis using data from the China Consortium of Reference Intervals Project (CHINRIP). This study employed a whole cluster, stratified random sampling method to collect 13,730 participants from six geographically diverse regions in China between January-July 2014. The age and sex composition and the urban/rural ratio of each community were determined by using the 2010 national census data of China (18). Inclusion criteria comprised: (i) subjects who were not pregnant; (ii) no administration of iodine-containing substances (including drugs and contrast media) within 3 months prior to enrollment and no current thyroid-directed pharmacotherapy; and (iii) no diagnosed developmental/psychiatric disorders. Fasting blood and spot urine samples were collected from each participant. Serum was obtained by centrifugation of the blood samples and stored at 4 °C prior to testing in the central laboratories of their respective city within 8 hours (Shanghai, Beijing, Guangzhou, Chengdu, Xi’an, and Shenyang in China). Participants underwent thyroid and liver ultrasonography for NAFLD, as well as an electrocardiogram (ECG) performed by trained and evaluated observers.

### Laboratory measurements

Free triiodothyronine (fT3), free thyroxine (fT4), TSH, thyroid peroxidase antibody (TPOAb), and thyroglobulin antibody (TgAb) levels were measured using an electrochemi-luminescence immunoassay (Cobas e601/E170, Roche Diagnostic, Indianapolis, IN, USA). All thyroid function parameters were analyzed as continuous variables, and no reference ranges were used for participant classification or exclusion. Lipid profiles, including serum triglyceride (TG), total cholesterol (TC), low-density lipoprotein cholesterol (LDL-C), high-density lipoprotein cholesterol (HDL-C) and serum uric acid (SUA), fasting plasma glucose (FBG) were measured by using Roche Modular automatic biochemical analyzer (Roche Diagnostics). Diagnostic criteria for metabolism and cardiovascular disorders are detailed in **Table 1** (19–29). Cardiovascular risk was quantified using Framingham Risk Score (FRS) incorporating age, sex, lipids, blood pressure, and smoking status (defined as >20 cigarettes/day) (30). Smoking status was assessed using a detailed questionnaire, defining regular smokers as those smoking more than 20 cigarettes per day.

### HPT sensitivity indices

Three indices were calculated. Firstly, the thyrotropin T4 resistance index (TT4RI) was derived by multiplying fT4 (pmol/L) with TSH (mIU/L). Secondly, the TSH index (TSHI) was determined using the following formula, ln TSH (mIU/L) + 0.1345 × fT4 (pmol/L). Lastly, the thyroid feedback quantile-based index (TFQI) is a new index calculated using the cumulative distribution function based on fT3 or fT4 and TSH (31).

The indices, TFQI, TSHI, and TT4RI, serve as indicators of central thyroid hormone sensitivity, providing insights into the HPT axis’s response to peripheral thyroid hormones (32).

### Statistical analysis

All statistical analyses were performed using R software, version 4.2.2 (R Foundation for Statistical Computing, Vienna, Austria), and MSTATA software (CRAN, Vienna, Austria). Continuous variables were reported as mean ± SD or median (IQR) based on distribution normality. Multivariable logistic regression analysis was adjusted for age (per 10-year increment), sex, body mass index (BMI), and outcome-specific covariates (e.g., SUA/FPG for metabolic outcomes). Nonlinear relationships between age and TFQI were examined using a linear regression model with Restricted cubic splines (RCS). By age (≤48 vs >48 years) based on RCS-derived inflection points, the data were divided into two distinct segments based on this inflection point.

### Ethical approval

The study protocols were approved by the Medical Ethics Committee of China Medical University (2012[53]). All participants provided informed consent before participating in the study.

## Results

### Clinical characteristics of subjects

The study enrolled 13,730 participants. After excluding 84 individuals due to incomplete information on thyroid function data, the final sample size was 13,646 participants. The participants had a mean age of 47 ± 15 years (range: 18–92 years), with 6,221 men and 7,425 women. The median (IQR) levels of fT3, fT4, and TSH were 4.84 (IQR: 4.45-5.28) pmol/L, 16.35 (IQR:15.0-17.8) pmol/L and 2.18 (IQR: 1.48-3.13) mIU/L, respectively. Mean (SD) values for BMI, UA, FPG, and non-HDL-C were 24.0 (3.7), 316 (89), 5.41 (1.25), and 3.41 (1.01). For creatine kinase (CK), creatine kinase myocardial band (CK-MB), and N-terminal pro-brain natriuretic peptide (NT-proBNP), the mean (SD) values were 125 (741), 1.64 (1.39), and 60 (149). Detailed diagnostic criteria for metabolism and cardiovascular disorders are provided in **Supplementary Table 1**. Participants were divided into four groups based on their HPT sensitivity IQR.

**Table 1** presents the clinical features of subjects in different HPT sensitivity groups. Age, sex, metabolic, and cardiovascular indicators were included. TFQI values range from -1 and 1, with positive values indicating poor thyroid hormone sensitivity, 0 indicating normal thyroid hormone sensitivity, and negative values indicating good sensitivity. Higher TSHI and TT4RI values correspond to lower central sensitivity to thyroid hormone. Individuals with lower HPT sensitivity (Q4) were younger and had a lower proportion of females compared to those with higher sensitivity (Q1). Metabolic indicators generally increased with decreased HPT sensitivity to fT3 (Q4) compared to normal or reduced sensitivity (Q1), a trend not observed for fT4 sensitivity.

**Table 1:** Clinical features of subjects in groups with different sensitivity to thyroid hormones.

	TFQI-fT3^1^ groups				P-value	TFQI-fT4^2^ groups				P-value
	Q1 (N = 3697)	Q2 (N = 3058)	Q3 (N = 3316)	Q4 (N = 3575)		Q1 (N = 3181)	Q2 (N = 3487)	Q3 (N = 3630)	Q4 (N = 3348)	
Age(mean ± SD)	49.81 ± 14.95	48.46 ± 14.69	47.05 ± 14.75	44.25 ± 14.80	<.001	48.90 ± 14.65	48.31 ± 14.46	47.33 ± 14.81	45.03 ± 15.59	<.001
Sex (%)										
male	1126 (30.5%)	1232 (40.3%)	1699 (51.2%)	2164 (60.5%)	<.001	1292 (40.6%)	1471 (42.2%)	1687 (46.5%)	1771 (52.9%)	<.001
female	2571 (69.5%)	1826 (59.7%)	1617 (48.8%)	1411 (39.5%)		1889 (59.4%)	2016 (57.8%)	1943 (53.5%)	1577 (47.1%)	
Metabolism index(mean ± SD)										
BMI	23.38 ± 3.64	23.92 ± 3.66	24.11 ± 3.60	24.61 ± 3.83	<.001	24.10 ± 3.72	24.18 ± 3.72	23.92 ± 3.70	23.80 ± 3.70	<.001
WHR	0.85 ± 0.08	0.86 ± 0.08	0.87 ± 0.07	0.89 ± 0.76	<.001	0.86 ± 0.07	0.86 ± 0.09	0.86 ± 0.16	0.87 ± 0.77	0.649
FPG	5.41 ± 1.38	5.39 ± 1.16	5.45 ± 1.30	5.41 ± 1.15	0.275	5.34 ± 1.11	5.43 ± 1.24	5.41 ± 1.22	5.46 ± 1.41	0.001
SUA	296 ± 85	310 ± 87	320 ± 90	335 ± 90	<.001	308.35 ± 91.83	311.25 ± 88.26	315.32 ± 88.58	327.27 ± 88.27	<.001
TG	1.30 ± 1.14	1.40 ± 1.24	1.46 ± 1.20	1.54 ± 1.15	<.001	1.42 ± 1.31	1.45 ± 1.28	1.42 ± 1.10	1.41 ± 1.04	0.431
TC	4.97 ± 1.03	4.90 ± 1.02	4.84 ± 0.98	4.75 ± 0.96	<.001	4.90 ± 1.04	4.90 ± 1.03	4.86 ± 0.98	4.80 ± 0.95	<.001
HDL	1.55 ± 0.40	1.46 ± 0.38	1.43 ± 0.39	1.37 ± 0.37	<.001	1.46 ± 0.38	1.45 ± 0.38	1.46 ± 0.40	1.45 ± 0.39	0.336
LDL	3.04 ± 0.91	3.03 ± 0.90	2.99 ± 0.88	2.94 ± 0.85	<.001	3.03 ± 0.92	3.04 ± 0.91	2.99 ± 0.87	2.94 ± 0.85	<.001
non-HDL	3.35 ± 0.98	3.39 ± 1.01	3.45 ± 1.03	3.45 ± 1.00	<.001	3.36 ± 1.02	3.41 ± 1.01	3.41 ± 1.01	3.45 ± 0.98	0.013
SBP	124± 19	125 ± 21	126 ± 17	126 ± 17	<.001	125 ± 18	126 ± 20	126 ± 18	126 ± 18	0.017
DBP	76 ± 10	77 ± 10	78 ± 10	78 ± 10	<.001	77 ± 11	78 ± 11	78 ± 11	78 ± 11	0.002
Cardiovascular Index(mean ± SD)										
HR	75 ± 10	76 ± 11	76 ± 11	76 ± 11	<.001	75 ± 10	76 ± 11	76 ± 11	76 ± 11	<.001
CK	133.62 ± 1362.59	111.14 ± 118.18	121.77 ± 185.37	131.32 ± 364.34	0.595	132.46 ± 1048.62	112.97 ± 116.22	132.73 ± 992.94	122.47 ± 331.73	0.643
CK-MB	1.59 ± 1.06	1.63 ± 1.35	1.69 ± 1.25	1.66 ± 1.79	0.015	1.77 ± 1.23	1.66 ± 1.37	1.65 ± 1.82	1.49 ± 0.95	<.001
NT-proBNP	73.90 ± 215.92	61.13 ± 115.80	53.63 ± 95.43	49.72 ± 125.73	<.001	60.60 ± 115.96	56.51 ± 105.45	59.10 ± 143.60	63.12 ± 207.96	0.313
hs-cTNI	0.00 ± 0.01	0.00 ± 0.01	0.00 ± 0.01	0.00 ± 0.01	0.787	0.00 ± 0.01	0.00 ± 0.01	0.00 ± 0.01	0.00 ± 0.01	0.893

^1^TFQI-fT3: Thyroid feedback quantile-based index by fT3 is calculated as cumulative distribution function (cdf) fT3-(1-cdf TSH).

^2^TFQI-fT4: Thyroid feedback quantile-based index by fT4 is calculated as cumulative distribution function (cdf) fT4-(1-cdf TSH). The population was divided into four parts based on the quartile ranges of TFQI.

BMI, body mass index; WHR, waist hip rate; FPG, fasting plasma glucose; SUA, serum uric acid; TG, triglyceride; TG, total cholesterol; LDL-C, low-density lipoprotein cholesterol; HDL-C, high-density lipoprotein cholesterol; SBP, systolic blood pressure; DBP, diastolic blood pressure; HR, heart rate; CK, creatine kinase; NT-proBNP, N-Terminal pro-brain natriuretic peptide; hs-cTNI, highly sensitive cardiac troponin I.

### Age-related changes in HPT sensitivity

**Figure 1:**
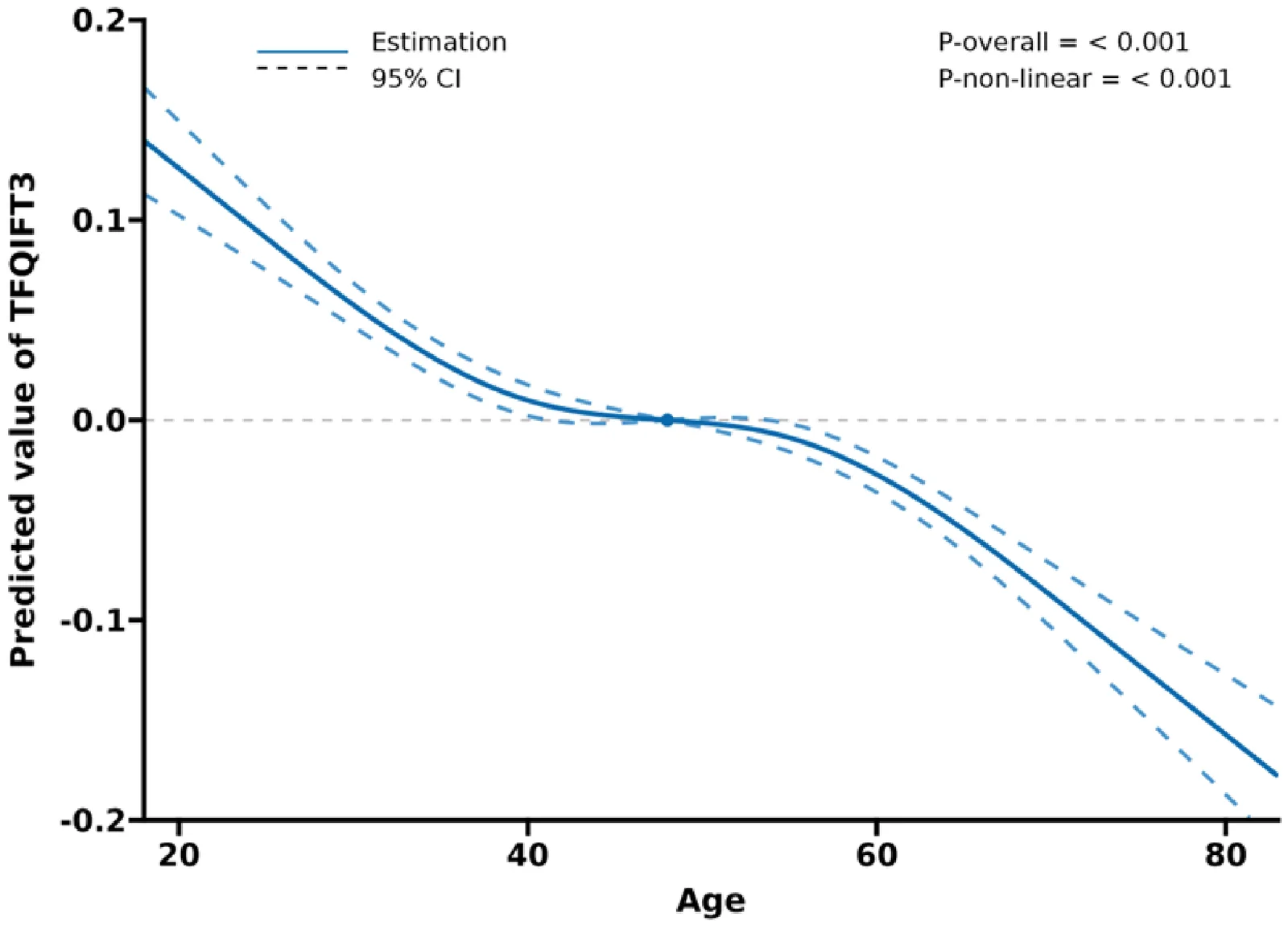
Association between age and TFQI-fT3 with the RCS function. TFQI-FT3: Thyroid feedback quantile-based index by fT3 is calculated as cumulative distribution function (cdf) fT3-(1-cdf TSH). The linear regression was adjusted for Sex. Y-axis represents the Predicted Values to present TFQI-fT3 for any value of Age compared to individuals with 48 of Age.

Age appears to correlate with thyroid hormone sensitivity (**Table 1**). To further explore this relationship, we analyzed thyroid hormone sensitivity across different age groups (**Table 2**). Results indicate that TFQI-fT3 significantly decreases with age, while TFQI-fT4 shows a similar but less pronounced trend. Trends for TT4RI and TSHI associated with age were unclear. We employed RCS analysis to examine the nonlinear association between age and TFQI-fT3, adjusting for sex and race (**Figure 1**). RCS analysis demonstrated a gradual decrease in TFQI-fT3 levels with increasing age, with values becoming negative after approximately 48 years. TFQI-fT3 was calculated as cdf(fT3) − [1 − cdf(TSH)], where cdf denotes the cumulative distribution function. A negative TFQI-fT3 value indicates that the percentile rank of TSH is lower than that of fT3 for a given individual. Hence, for a given fT3 level, TSH is more suppressed relative to the population distribution. Since a lower fT3 signal produces a greater relative suppression of TSH secretion, this pattern reflects enhanced feedback sensitivity of the HPT axis to fT3. Therefore, the observed age-related decline in TFQI-fT3 is interpreted as an age-dependent increase in central sensitivity to fT3. This age-related enhancement of HPT axis sensitivity to fT3 may represent a physiological adaptation to maintain metabolic homeostasis during aging. The same logic also applies to TFQI-fT4.

**Table 2:** Sensitivity to thyroid hormones indexes in different age groups.

	Age group (years)							P-value
	18-29	30-39	40-49	50-59	60-69	70-79	≥80	
	N = 2,122	N = 2,403	N = 2,742	N = 3,056	N = 2,373	N = 896	N = 54	
TFQI-fT3^1^								<0.001
Median (IQR)	0.10 (-0.15, 0.35)	0.05 (-0.18, 0.28)	0.00 (-0.24, 0.24)	0.00 (-0.24, 0.22)	-0.04 (-0.27, 0.19)	-0.09 (-0.35, 0.14)	-0.18 (-0.36, 0.06)	
TFQI-fT4 ^2^								<0.001
Median (IQR)	0.09 (-0.14, 0.34)	0.03 (-0.18, 0.25)	-0.02 (-0.24, 0.19)	0.00 (-0.23, 0.22)	-0.01 (-0.21, 0.19)	-0.02 (-0.26, 0.20)	-0.01 (-0.17, 0.24)	
TT4RI^3^								<0.001
Median (IQR)	37 (26, 51)	36 (25, 50)	34 (24, 49)	35 (24, 51)	37 (24, 54)	35 (23, 56)	39 (27, 71)	
TSHI^4^								<0.001
Median (IQR)	3.08 (2.69, 3.45)	3.01 (2.63, 3.37)	2.94 (2.55, 3.31)	2.98 (2.55, 3.36)	2.99 (2.59, 3.39)	2.97 (2.54, 3.42)	3.03 (2.65, 3.65)	

^1^TFQI-fT3: Thyroid feedback quantile-based index by fT3 is calculated as cumulative distribution function (cdf) fT3-(1-cdf TSH).

^2^TFQI-fT4: Thyroid feedback quantile-based index by fT4 is calculated as cumulative distribution function (cdf) fT4-(1-cdf TSH). The population was divided into four parts based on the quartile ranges of TFQI.

^3^TT4RI: Thyrotropin T4 resistance index was derived by multiplying fT4 (pmol/L) with TSH (mIU/L).

^4^TSHI: The TSH index was determined as ln TSH (mIU/L) + 0.1345 · fT4 (pmol/L).

### HPT sensitivity and metabolic and cardiovascular disorders

**Figure 2:**
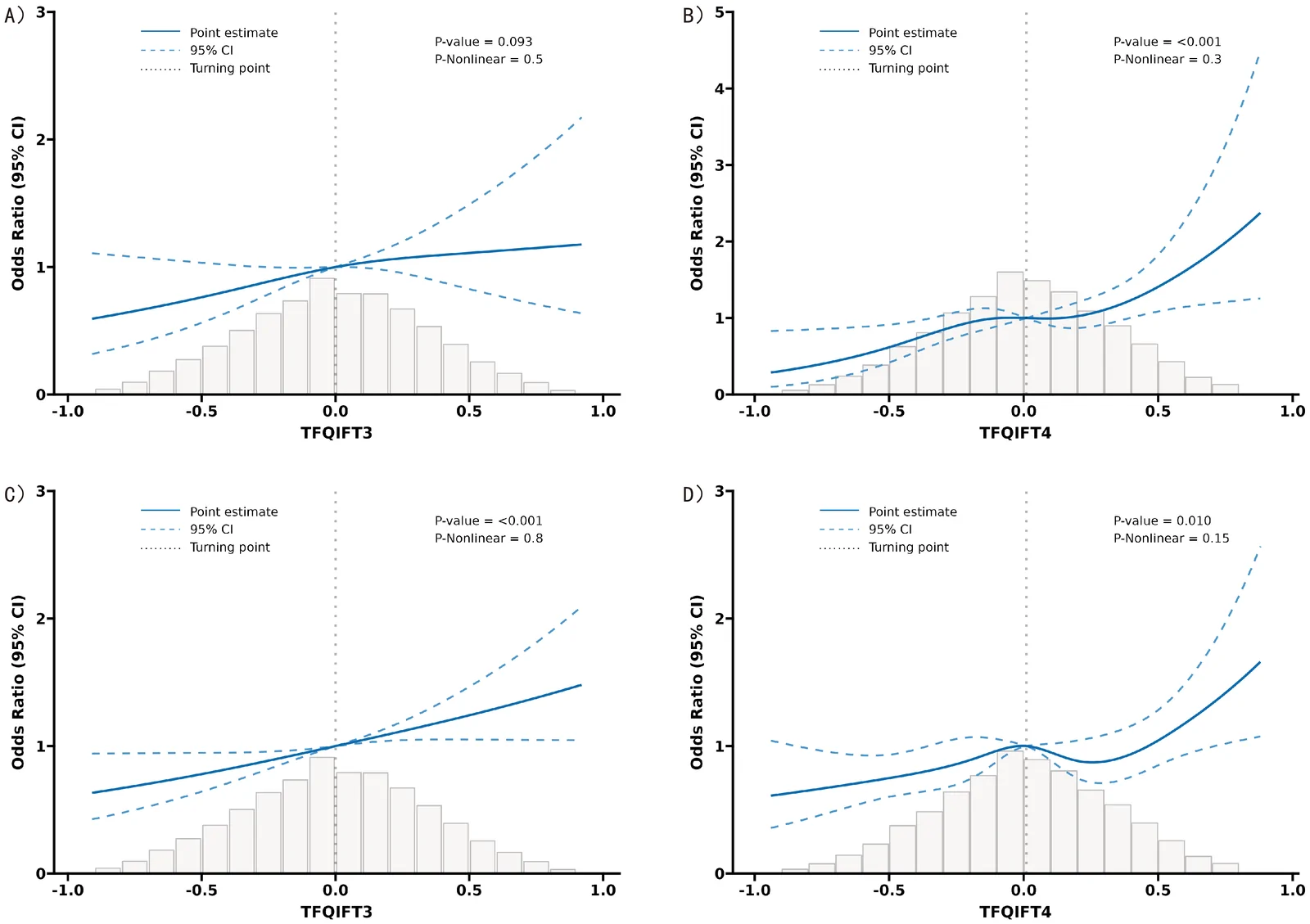
Restricted cubic spline (RCS) analyses of associations between TFQI indices and abnormal ST segment or metabolic syndrome (MetS). **(A–D)** show the RCS curves for each combination of TFQI index and outcome, adjusted for age and sex. Solid lines represent odds ratios, and shaded areas indicate 95% confidence intervals. P values for overall and nonlinear tests are shown in each panel. **(A)** Association between TFQI-fT3 and abnormal.ST with the RCS function. **(B)** Association between TFQI-fT4 and abnormal.ST with the RCS function. **(C)** Association between TFQI-fT3 and MetS with the RCS function. **(D)** Association between TFQI-fT4 and MetS with the RCS function.

To analyze the impact of TFQI on metabolic and cardiovascular disorders, we used logistic regression for multivariable analysis in adults. **Table 3** shows that in the older adults (aged over 48 years), increased HPT sensitivity to fT3 (TFQI-fT3 Q1) is a protective factor against metabolic syndrome (MetS) (OR 0.79, p=0.023). However, this trend was not statistically significant in the adult population or for HPT sensitivity to fT4. **Table 4** reveals that increased HPT sensitivity to fT3 (TFQI-fT3 Q1) is associated with a decreased risk of abnormal ST segment in both younger adults (OR 0.57, p=0.046) and older adults (aged over 48 years) (OR 0.68, p=0.027). In contrast, while increased HPT sensitivity to fT4 (TFQI-fT4 Q1) was not significantly associated, decreased HPT sensitivity to fT4 (TFQI-fT4 Q4) was a risk factor for abnormal ST segment in adults (OR 1.43, p=0.025). Logistic regression analyses for TT4RI and TSHI showed similar but generally weaker associations compared with TFQI. Detailed results are presented in **Supplementary Tables 2** and **3**. RCS analyses, adjusted for age and sex, were performed to visualize the shape of the associations between TFQI indices and outcomes (**Figure 2**). As the P values for nonlinearity were not significant across all panels, we used the quartile-based TFQI groups (**Tables 3**, **4**) as the primary analytic approach to avoid imposing a specific functional form.

**Table 3:** Multivariable analysis of influencing factors for metabolic syndrome (MetS) using logistic regression in adult (≤48 years old) and elderly (>48 years old) population.

Population	TFQI groups^2^	TFQI-fT3 model for MetS^1^					TFQI-fT4 model for MetS^1^				
		N	Event N	OR^3^	95% CI^3^	p-value	N	Event N	OR^3^	95% CI^3^	P-value
Younger Adults^4^ (≤48 years old)	Reference	3,228	122	—	—		3,586	146	—	—	
	Increased	1,674	42	0.99	0.68, 1.42	0.956	1,531	57	1.03	0.75, 1.42	0.835
	Decreased	2,108	113	1.17	0.89, 1.53	0.254	1,893	74	0.87	0.64, 1.16	0.335
Older Adults^5^ (>48 years old)	Reference	3,146	329	—	—		3,531	355	—	—	
	Increased	**2,023**	**165**	**0.79**	**0.65, 0.97**	0.023	1,650	147	0.85	0.69, 1.04	0.109
	Decreased	1,467	173	1.11	0.91, 1.35	0.297	1,455	165	1.13	0.92, 1.37	0.236

^1^Multivariable logistic regression analysis models adjusted for potential factor including age (10 years), sex, TFQI-fT3(or TFQI-fT4) groups with the outcome of Metabolic Syndrome (MetS).

^2^TFQI groups are defined by first dividing the data into four quartiles, denoted as Q1, Q2, Q3, and Q4, based on the interquartile range. The groups are then classified as follows: Q1 is labeled as the “Increase” group, representing the lowest quartile. Q2 and Q3 are combined and labeled as the “Reference” groups. Q4 is labeled as the “Decrease” group, representing the highest quartile.

^3^OR, Odds Ratio; CI, Confidence Interval.

^4^Number in data frame = 7010, Number in model = 7010, Missing = 0, AIC = 2131.9, C-statistic = 0.744, H&L = Chi-sq (8) 2.49 (p=0.962).

^5^Number in data frame = 6636, Number in model = 6636, Missing = 0, AIC = 4198.9, C-statistic = 0.64, H&L = Chi-sq (8) 18.06 (p=0.021).

Bold values indicate statistical significance (p < 0.05).

**Table 4:** Multivariable analysis of influencing factors for abnormal ST segment using logistic regression in adult (≤48 years old) and elderly (>48 years old) population.

Population	TFQI groups^2^	TFQI-fT3 model for abnormal ST segment^1^					TFQI-fT4 model for anormal ST segment^1^				
		N	Event N	OR^3^	95% CI^3^	p-value	N	Event N	OR^3^	95% CI^3^	P-value
Younger Adults^4^ (≤48 years old)	Reference	3,225	49	—	—		3,581	47	—	—	
	Increased	**1,672**	**18**	**0.57**	**0.32, 0.97**	**0.046**	1,530	16	0.72	0.39, 1.25	0.256
	Decreased	2,103	19	0.75	0.43, 1.27	0.301	1,889	23	1.11	0.65, 1.82	0.693
Older Adults^5^ (>48 years old)	Reference	3,136	106	—	—		3,521	112	—	—	
	Increased	**2,019**	**52**	**0.68**	**0.48, 0.95**	**0.027**	1,649	37	0.7	0.47, 1.01	0.063
	Decreased	1,463	55	1.25	0.89, 1.73	0.198	**1,448**	**64**	**1.43**	**1.04, 1.96**	0.025

^1^Multivariable logistic regression analysis models adjusted for potential factor including age (10 years), sex, BMI, SUA, FPG, non-HDL, TFQI-fT3(or TFQI-fT4) groups with the outcome of abnormal ST segment.

^2^TFQI groups are defined by first dividing the data into four quartiles, denoted as Q1, Q2, Q3, and Q4, based on the interquartile range. The groups are then classified as follows: Q1 is labeled as the “Increase” group, representing the lowest quartile. Q2 and Q3 are combined and labeled as the “Reference” groups. Q4 is labeled as the “Decrease” group, representing the highest quartile.

^3^OR, Odds Ratio; CI, Confidence Interval.

^4^Number in data frame=6636, Number in model=6636, Missing=0, AIC = 4202.2, C-statistic=0.638, H&L=Chi-sq (8) 18.52 (p=0.018).

^5^Number in data frame=6636, Number in model=6618, Missing=18, AIC = 1844.1, C-statistic=0.659, H&L=Chi-sq (8) 8.35 (p=0.400).

Bold values indicate statistical significance (p < 0.05).

## Discussion

Our population-based study identifies a age-dependent enhancement in HPT axis sensitivity to fT3, with distinct metabolic and cardiovascular correlations. Three key findings were promoted: (1) TFQI-fT3 demonstrates a nonlinear decline with age (inflection at 48 years); (2) Increased fT3 sensitivity is associated with a lower prevalence of MetS and ST-abnormality risks in adults >48 years; (3) This association pattern exhibits hormone specificity, as fT4 sensitivity showed opposing cardiovascular effects. These observations extend current understanding of thyroid aging by integrating HPT axis dynamics with clinical outcomes.

Circulating thyroid hormones are synthesized and released by the thyroid gland, regulated by the HPT axis. The HPT axis regulates thyroid hormone homeostasis through a classic feedback loop including hypothalamic thyrotropin-releasing hormone (TRH) stimulates pituitary TSH secretion, which promotes thyroidal release of T4 and T3 (33). T3’s activity is about ten times higher than T4’s, and T4 can be converted to T3 by deiodinases, iodothyronine deiodinase type 1(DIO1) and DIO2, in extra-thyroidal tissues. DIO1, primarily active in the liver and kidneys, generates 15-20% of the total circulating T3 (34), while DIO2, found in brown adipose tissue, the pituitary gland, the brain, and the heart, is responsible for the majority of T3 production (35).

Thyroid hormones regulate metabolic processes and maintain homeostasis within their normal range. They significantly impact basal metabolic rate, glucose absorption, lipolysis, and serum cholesterol levels (36–38). These hormones also affect the cardiovascular system by modulating endothelial cell function, heart rate, and myocardial oxygen consumption (39). Clinical thyroid dysfunctions, such as hyperthyroidism and hypothyroidism, are strongly associated with obesity, dyslipidemia, atrial fibrillation, and other cardiovascular diseases (CVDs) (40, 41). Subclinical thyroid conditions, often without overt symptoms, also carry significant risks for CVD and metabolic disorders. Studies show that subclinical hypothyroidism is associated with vascular abnormalities like increased systemic vascular resistance and altered endothelial-mediated vasorelaxation and vascular compliance (42, 43). Subsequent evidence links impaired sensitivity to hyperuricemia, abdominal fat, hypertension, and diabetes (32, 44–46). Our results corroborate Laclaustra et al. (2019) report linking impaired thyroid sensitivity (higher TFQI) to metabolic dysfunction (31), but uniquely identify an age-dependent protective effect of fT3 sensitivity. The lower MetS risk (OR 0.79) in high-sensitivity older adults (>48 years) may reflect optimized energy homeostasis during aging. Which is the hypothesis supported by animal studies showing DIO2 upregulation preserves cardiac function in aged mice (47).

The age-related TSH rise with stable/falling T3 levels has been interpreted variably - as subclinical hypothyroidism or adaptive response (48, 49). Recent studies found that the set point of the HPT axis and peripheral sensitivity to thyroid hormones vary across different age groups (31). In our study, TFQI-fT3 significantly decreases with increasing age, indicating that normal low levels of fT3 are more likely to cause elevated TSH during aging, indirectly explaining higher TSH levels in the old adults. The RCS-derived inflection point (48 years) is an exploratory finding from this study, suggesting that midlife may represent a potential transition period in thyroid regulation. However, this specific threshold requires validation in independent external studies.

Mild evaluated TSH levels are considered a sign of healthy aging in some studies. For example, patients aged 85–89 with elevated TSH had lower mortality (hazard ratio 0.77, 95% CI 0.63–0.94) (50), and patients aged 70–79 with TSH values between 4.5 and 7 mIU/L showed improved mobility (51). Rakov et al. revealed that aging induces organ-specific hypersensitivity to T4 despite systemic T3 decline, suggesting central and peripheral mechanisms may compensate for reduced thyroid output (52). Our TFQI-fT3 findings extend this pattern by showing enhanced central sensitivity to fT3 specifically is associated with favorable metabolic and cardiovascular profiles in older adults. In the older adults (aged over 48 years), increased HPT sensitivity to fT3 (TFQI-fT3 Q1) was associated with lower odds of MetS (OR 0.79, p = 0.023) and a lower prevalence of abnormal ST segments. Subclinical hypothyroidism and its risk of CVDs are controversial, especially in the elderly. Some studies show that treating persistent subclinical hypothyroidism (TSH 5·0-10·0 mIU/L) reduces cardiovascular events in adults, but not in those over 70 (53). Our results suggest that higher HPT axis sensitivity to fT3 is associated with lower prevalence of metabolic and cardiovascular conditions.

The main strengths of our study lie in (1) Nationally representative sampling (n=13,646) with standardized sample collection; (2) Novel application of TFQI to characterize aging HPT sensitivity changes and its association with metabolic/cardiovascular outcomes. However, our study has limitations. Although the data were collected between 2012 and 2014, the core biological associations examined in this study are expected to be stable over time. Nevertheless, the cross-sectional design and the age of the data should be considered when interpreting the findings. Additionally, the 48-year age cutoff used for stratification was identified from the same dataset using exploratory restricted cubic spline analysis. Its generalizability requires validation in independent external studies, and the stratified results should be interpreted with appropriate caution. Furthermore, multicenter investigations across different regions face potential impacts from geographic, environmental, and dietary factors, which may influence disease outcomes. Finally, as a cross-sectional survey, this study cannot determine the precise underlying mechanisms linking aging, thyroid function, metabolic disorders, and CVD risk, and thus future prospective studies are needed.

## Data Availability

The raw data supporting the conclusions of this article will be made available by the authors, without undue reservation.

## Supplementary material

Supplementary Table 1 Diagnostic criteria for metabolism and cardiovascular disorders. BMI, body mass index; WHR, waist hip rate; FPG, fasting plasma glucose; SUA, serum uric acid; TG, triglyceride; TG, total cholesterol; LDL-C, low-density lipoprotein cholesterol; HDL-C, high-density lipoprotein cholesterol; SBP, systolic blood pressure; DBP, diastolic blood pressure; HR, heart rate; CK, creatine kinase; NT-proBNP, N-Terminal pro-brain natriuretic peptide; hs-cTNI, highly sensitive cardiac troponin I.

Supplementary Table 2 Multivariable analysis of TT4RI and TSHI for Metabolic Syndrome (MetS) using logistic regression. 1: Multivariable logistic regression analysis models adjusted for potential factor including age (10 years), sex, BMI and TT4RI (or TSHI) groups with the outcome of Metabolic Syndrome (MetS). 2: TT4RI (or TSHI) groups are defined by first dividing the data into four quartiles, denoted as Q1, Q2, Q3, and Q4, based on the interquartile range. The groups are then classified as follows: Q1 is labeled as the “Increase” group, representing the lowest quartile. Q2 and Q3 are combined and labeled as the “Reference” groups. Q4 is labeled as the “Decrease” group, representing the highest quartile. 3: OR = Odds Ratio, CI = Confidence Interval.

Supplementary Table 3 Multivariable analysis of TT4RI and TSHI for abnormal ST Segment using logistic regression. 1: Multivariable logistic regression analysis models adjusted for potential factor including age (10 years), sex, BMI, SUA, FPG, non-HDL and TT4RI (or TSHI) groups with the outcome of abnormal ST segment. 2: TT4RI (or TSHI) groups are defined by first dividing the data into four quartiles, denoted as Q1, Q2, Q3, and Q4, based on the interquartile range. The groups are then classified as follows: Q1 is labeled as the “Increase” group, representing the lowest quartile. Q2 and Q3 are combined and labeled as the “Reference” groups. Q4 is labeled as the “Decrease” group, representing the highest quartile. 3: OR=Odds Ratio, CI=Confidence Interval.
