# Supplementary material for: Age-related changes in HPT axis sensitivity and their associations with metabolic and cardiovascular health: a cross-sectional, Chinese nationwide study
Source: Front Endocrinol (Lausanne). 2026 Sep 9;17:1857191. doi: 10.3389/fendo.2026.1857191 (PMC13597420; doi:10.3389/fendo.2026.1857191)
Supplement: Supplementary file 1 [file DataSheet1.docx]

Supplementary table 1: Diagnostic Criteria for metabolism and cardiovascular disorders

| Disorders | Diagnostic Criteria |
| --- | --- |
| Obesity^(19)^ | BMI≥28 kg/m^2^ |
| Central Obesity^(19)^ | weight circumference male≥90cm female≥85cm |
| Dyslipidemia^(21)^ | TC≥6.5 mmol/l and/or TG≥1.7 mmol/l and/or LDL≥3.4 mmol/l and/or HDL<1.0mmol/l |
| Hyperuricemia^(20)^ | male and female  SUA>405mmol/l |
| Impaired Fasting Glucose(IFG)^(51)^ | Fasting glucose≥6.1mmol/l |
| Hypertension^(52)^ | SBP≥140mmHg and/or DBP≥90mmHg |
| Metabolic Syndrome(MetS)^(25)^ | any 3 of 5 constitute diagnosis MetS including central obesity, dyslipidemia (evaluated triglycerides or reduced HDL-C), hypertension and elevated fasting glucose |
| Non-alcoholic fatty liver disease(NAFLD)^(53)^ | diagnosed by liver ultrasound radiologist |
| Abnormal ST^(26)^ | ST-segment depression ≥0.5 mm in depth or elevation ≥2 mm in two or more contiguous leads |

Abbreviations: BMI, body mass index; WHR, waist hip rate; FPG, fasting plasma glucose; SUA, serum uric acid; TG, triglyceride; TG, total cholesterol; LDL-C, low-density lipoprotein cholesterol; HDL-C, high-density lipoprotein cholesterol; SBP, systolic blood pressure; DBP, diastolic blood pressure; HR, heart rate; CK, creatine kinase; NT-proBNP, N-Terminal pro-brain natriuretic peptide; hs-cTNI, highly sensitive cardiac troponin I.

Supplementary table 2: Multivariable analysis of TT4RI and TSHI for Metabolic Syndrome (MetS) using logistic regression

|  |  | **TT4RI Model for MetS^1^** | | | | | **TSHI Model for MetS^1^** | | | | |
| --- | --- | --- | --- | --- | --- | --- | --- | --- | --- | --- | --- |
| **Population** | **Sub- groups^2^** | **N** | **Event N** | **OR^3^** | **95% CI^3^** | **p-value** | **N** | **Event N** | **OR^3^** | **95% CI^3^** | **p-value** |
| Whole population | Reference | 6,801 | 467 | 1 | - | - | 6,800 | 483 | — | — |  |
|  | Increased | 3,399 | 231 | 0.9 | 0.76-1.07 | 0.23 | 3,342 | 218 | 0.87 | 0.73, 1.03 | 0.11 |
|  | Decreased | 3,399 | 244 | 1.07 | 0.90-1.26 | 0.465 | 3,457 | 241 | 1.03 | 0.87, 1.22 | 0.749 |

1: Multivariable logistic regression analysis models adjusted for potential factor including age (10 years), sex, BMI and TT4RI (or TSHI) groups with the outcome of Metabolic Syndrome (MetS).

2: TT4RI (or TSHI) groups are defined by first dividing the data into four quartiles, denoted as Q1, Q2, Q3, and Q4, based on the interquartile range. The groups are then classified as follows: Q1 is labeled as the "Increase" group, representing the lowest quartile. Q2 and Q3 are combined and labeled as the "Reference" groups. Q4 is labeled as the "Decrease" group, representing the highest quartile.

3: OR = Odds Ratio, CI = Confidence Interval.

Supplementary table 3: Multivariable analysis of TT4RI and TSHI for Abnormal ST Segment using logistic regression

|  |  | **TT4RI Model for abnormal ST segment^1^** | | | | | **TSHI Model for anormal ST segment^1^** | | | | |
| --- | --- | --- | --- | --- | --- | --- | --- | --- | --- | --- | --- |
| **Population** | **Sub- groups^2^** | **N** | **Event N** | **OR^3^** | **95% CI^3^** | **p-value** | **N** | **Event N** | **OR^3^** | **95% CI^3^** | **p-value** |
| Whole population | Reference | 6,809 | 135 | 1 | - | - | 6,808 | 165 | 1 | - | - |
|  | Increased | 3,401 | 57 | 0.81 | 0.59-1.11 | 0.197 | **3,344** | **57** | **0.68** | **0.50-0.92** | **0.014** |
|  | Decreased | **3,403** | **106** | **1.38** | **1.06-1.79** | **0.015** | 3,461 | 76 | 0.93 | 0.70-1.23 | 0.605 |

1: Multivariable logistic regression analysis models adjusted for potential factor including age (10 years), sex, BMI, SUA, FPG, non-HDL and TT4RI (or TSHI) groups with the outcome of abnormal ST segment.

2: TT4RI (or TSHI) groups are defined by first dividing the data into four quartiles, denoted as Q1, Q2, Q3, and Q4, based on the interquartile range. The groups are then classified as follows: Q1 is labeled as the "Increase" group, representing the lowest quartile. Q2 and Q3 are combined and labeled as the "Reference" groups. Q4 is labeled as the "Decrease" group, representing the highest quartile.

3: OR=Odds Ratio, CI=Confidence Interval.
